# Early Reconstruction Delivered Better Outcomes for Severe Open Fracture of Lower Extremities: A 15-Year Retrospective Study

**DOI:** 10.3390/jcm11237174

**Published:** 2022-12-02

**Authors:** Zheming Cao, Cheng Li, Jiqiang He, Liming Qing, Fang Yu, Panfeng Wu, Juyu Tang

**Affiliations:** Division of Hand and Microsurgey, Department of Orthopedic, Xiangya Hospital of Central South University, Changsha 410008, China

**Keywords:** open fracture, orthoplastic surgery, reconstruction, flap surgery, perforator flap

## Abstract

Background: The principle of early flap reconstruction for high-grade traumatic lower-extremity injuries established in 1986 by Godina has been widely accepted. However, the lack of an orthoplastic center in China makes early reconstruction not accessible for all patients with a Gustilo IIIB fracture. This study aimed to analyze the impact of timing on outcomes in lower-extremity traumatic free-flap reconstruction. Methods: A retrospective review identified 394 free-flap reconstructions performed from January 2005 to January 2020 for Gustilo IIIB tibial fractures. Patients were stratified based on the number of debridements: two times or less (early) and more than two times (delayed). The interval between injury and reconstruction, surgery time, hemorrhage volume, length of hospitalization (LoS), wound and bone healing time, flap outcomes, and function restoration were examined based on times of debridement. Results: The mean interval between injury and flap reconstruction in the early-repair group with 6.15 ± 1.82 postoperative days (PODs) was significantly shorter than that of the delayed-repair group with 16.46 ± 4.09 PODs (*p <* 0.001). The flap harvest time, reconstructive time, and intraoperative blood loss were also significantly less in the early-repair group compared to the delayed-repair group. Interestingly, we observed an 8.20% enlargement of wound size due to multiple debridements in the delayed-repair group. Most importantly, the early-repair group had better outcomes with a decreased risk of total or partial flap necrosis, lower incidence of flap complications, and fewer overall late complications than the delayed-repair group. In addition, the LoS, as well as wound and bone healing time, were notably shorter in the early-repair group. Furthermore, 4.85% of cases in the delayed-repair group experienced additional operations on bone, while no additional operations were performed in the early-repair group. All cases in both groups obtained satisfying functional results, while the early-repair group showed better functional recovery. Conclusions: Early repair with free flaps performed within two instances of debridement had superior outcomes when compared with delayed reconstruction after multiple debridements, consistent with Godina’s findings. We recommended early referral to a higher-level hospital with orthoplastic capabilities after an aggressive and thorough initial debridement carried out by senior surgeons.

## 1. Introduction

Owing to the fast-growing industrialization, motorization, and aging of the population, the burden of open fractures has increased tremendously [1,2]. It is estimated that 4.39 million fractures occurred in China in 2014, with 3–6 million people experiencing a fracture in the United States annually, 3% of which are open fractures [3,4]. Open fractures tend to cause extensive damage to the bone and overlying soft tissue, and the severity is generally assessed by the Gustilo-Anderson open fractures classification system, which categorizes fractures by wound size, contamination, and soft-tissue injury [5,6]. The standard treatment of open fractures includes systemic and local antibiotic prophylaxis to prevent infection, thorough debridement to remove devitalised tissue, surgical intervention to repair vascular and tissue damage and stabilize fractures with either internal or external fixation, and soft-tissue coverage with early or delayed reconstruction [7,8,9,10]. Timely soft-tissue coverage prevents bone and soft tissue desiccation, accelerates healing, and minimalizing infection.

The timing of soft-tissue coverage is critical to long-term outcomes in open fractures, although it remains a controversial topic. Over the past few decades, several studies have demonstrated that early soft-tissue coverage of the traumatic wound ranging from three days to 15 days decreased complications and improved reconstructive outcomes [11,12,13,14,15]. Growing evidence indicates that soft-tissue closure or coverage within 72 h of injury delivers satisfactory outcomes, indicating that multidisciplinary co-operative treatment is crucial to optimizing outcomes and that the early intervention of a collaborative orthoplastic approach is desirable for the management of open fractures [10,14,16,17,18,19]. Typically, based on the strength of orthopedic stable bone fixation and well vascularized coverage by plastic flaps, orthoplastic approaches lead to accelerated bone union, reliable soft-tissue coverage, shorter hospital stays, fewer complications and revision surgeries, and higher patient satisfaction [19,20,21]. However, it is impractical to perform early flap reconstruction in all cases of open fractures, especially in patients with concomitant injuries and underlying medical conditions.

On the contrary, there are some publications indicating that the delay of free flap coverage did not deliver worse outcomes, even when reconstruction was performed 90 days after injury [22,23,24,25]. With modern advancements in debridement and irrigation devices, broad-spectrum antibiotic coverage, and wound-management methods, the outcomes of delayed reconstruction might be guaranteed. Here, the impact of the duration preceding definitive free flap coverage on reconstructive outcomes in patients with severe lower-extremity open fractures was statistically analyzed.

## 2. Patients and Methods

### 2.1. Patients

This retrospective study enrolled 394 cases (234 males and 160 females) of Gustilo IIIB tibial fractures, admitted to our hospital between January 2005 and January 2020, which were divided into two groups based on the number of debridements: the early-repair group (debridement ≤ 2 times, N = 167) and the delayed-repair group (debridement > 2 times, N = 227). The same surgical team performed all the procedures. This study followed the guidelines of the Medical Ethics Committee of Xiangya Hospital, Central South University, and the ethical standards of the Helsinki Declaration of 1975, and all subsequent revisions.

The inclusion criteria are as follows: (1) Gustilo IIIB tibial fractures without segmental bone loss; (2) emergency trauma admitted to this medical center and referral to this medical center after debridement in other hospitals; (3) soft-tissue defects repaired by latissimus dorsal musculocutaneous flap (LD), anterolateral thigh perforator flap (ALTP), or both; (4) a follow-up time longer than 2 years. The exclusion criteria are as follows: (1) nontraumatic etiologies, such as diabetic ulceration or neoplasm; (2) inadequate medical record available for review, including cases in which a previous surgery or other conditions could affect the clinical results; (3) multiple-staged flap reconstruction due to over-large wound size.

### 2.2. Surgical Technique

**Figure 1:**
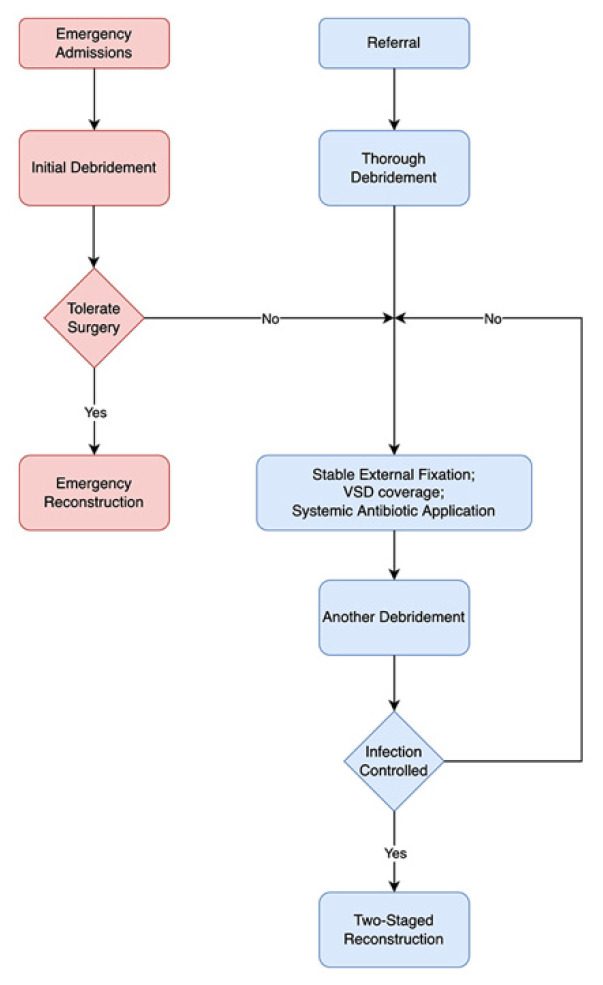
The flow diagram of two reconstructive patterns. For emergency admissions who could tolerate the surgery, emergency soft-tissue repair was conducted after thorough debridement. For referral and emergency admissions who could not tolerate surgical access, thorough debridement was performed first, and the flap coverage time was evaluated according to the condition of the wound.

**Figure 2:**
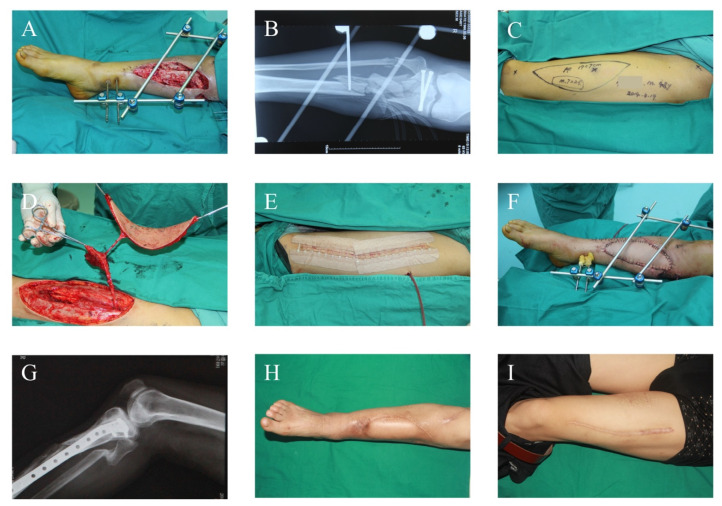
Early-Repair Case Report. A 48-year-old male patient with a Gustilo IIIB open fracture due to a traffic accident was admitted to our emergency department. (**A**,**B**) The condition after initial radical debridement. (**C**,**D**) The designed contralateral ALTP and harvest of chimeric ALTP. (**E**) The donor site was closed directly. (**F**) The soft-tissue coverage of recipient site. (**G**) The internal fixation was applied after 3 months of emergency flap surgery. (**H**,**I**) The condition of recipient and donor site 9 months postoperative. Only a linear scar remained in the donor site. ALTP: anterolateral thigh perforator flap.

**Figure 3:**
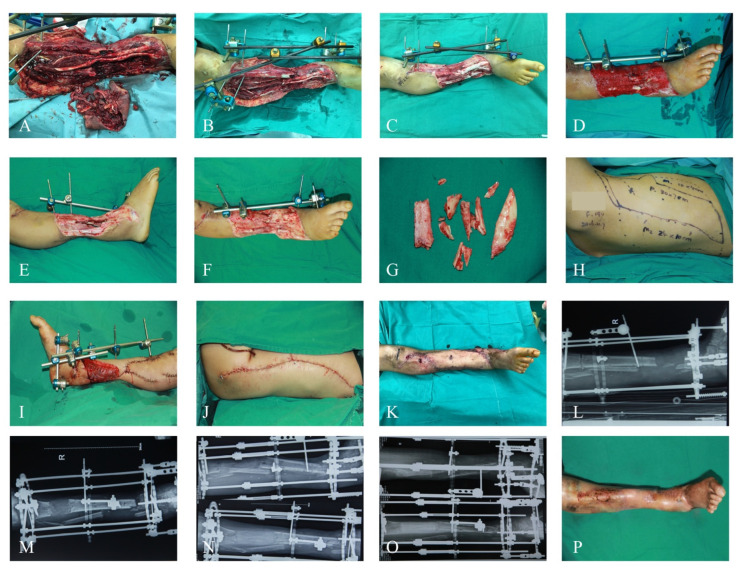
Delayed-Repair Case Report. A 19-year-old male patient with several lower-extremity injuries after a traffic accident was admitted to our emergency department. (**A**) The condition at admission. (**B**) The condition after initial radical debridement. (**C**) The condition 14 days after injury, after third debridement. (**D**–**G**) The condition 21 days after injury. (**D**) The granulation tissue condition after VSD removal. (**E**,**F**) The condition after fourth debridement. (**G**) The removed of devitalized bone tissue. (**H**) The designed ipsilateral LD. (**I**) The soft-tissue coverage of the recipient site. (**J**) The donor site was closed directly. (**K**) The condition two months after flap reconstruction, with external fixation removed and Ilizarov frame applied. (**L**–**O**) The radiographic view 1 week, 3 months, 6 months, and 12 months after Ilizarov lengthening. (**P**) The condition after two years of injury. VSD: vacuum closed drainage; LD: latissimus dorsi musculocutaneous flap.

Different surgical patterns were conducted depending on the condition of the patients (Figure 1). For emergency admissions that could tolerate the surgery, which was assessed by experienced consultants, emergency soft-tissue repair was conducted after thorough debridement (Figure 2). For referral and emergency admissions who could not tolerate surgical access, thorough debridement was performed first, and the flap coverage time was evaluated according to the condition of the wound (Figure 3). After admission, the necrotic tissues were meticulously and thoroughly removed by experienced microsurgeons. The specimens were taken for bacterial culture and drug sensitivity to guide antibiotic choice, the defects were covered by vacuum closed drainage (VSD), and the fracture was stabilized by external fixation. The number of debridements was determined according to the contamination of the wound. ALTP, LD, or combined flaps were used to cover large soft-tissue defects.

The following outcomes were evaluated in accordance with the time interval before reconstruction: (1) free flap complications; (2) risk factors of flap necrosis; (3) donor site morbidity; (4) long-term complications, including deep infection and osteomyelitis; and (5) additional operations to bone. The operative time for flap harvest and reconstruction, hemorrhage volume during reconstruction, length of hospitalization (LoS), and wound and bone healing time were also evaluated. A modified Enneking score was used to evaluate the functional recovery of the lower extremity in all patients 3, 6, 9, 12, and 24 months postoperative.

### 2.3. Statistical Analysis

SPSS Version 21.0 (IBM Corp., Armonk, NY, USA) and GraphPad Prism Version 5.0 (GraphPad Software, San Diego, CA, USA) were used for statistical analysis and plotting graphs, respectively. Quantitative data was expressed as means ± standard deviation and compared using the Student’s *t*-test or one-way ANOVA-LSD test. The paired *t*-test was applied to analyze wound size enlargement in the delayed-repair group. Qualitative data was expressed as numbers or percentages and compared using the χ^2^ test and Fisher’s exact test. A value of *p* < 0.05 was considered statistically significant.

## 3. Results

A total of 394 patients ranging in age from 7 to 64 years, with variable defect sizes of 102.5 to 470 cm^2^, were included in the present study. No significant difference was observed in terms of age, sex, BMI, alcohol consumption, tobacco and nicotine use, chronic diseases, trauma mechanism, location of wounds, or soft-tissue defects size between the early-repair and delayed-repair groups. The homogeneity of the two groups was good, and the results pertaining to short- and long-term follow-ups could be considered comparable (Table 1). While patients in the early-repair group were admitted mainly through the emergency department, the majority of patients in the delayed-repair group were referred from other hospitals. The rates of wound infection and coagulation abnormity were also higher in the delayed-repair group than in the early-repair group (Table 1).

**Figure 4:**
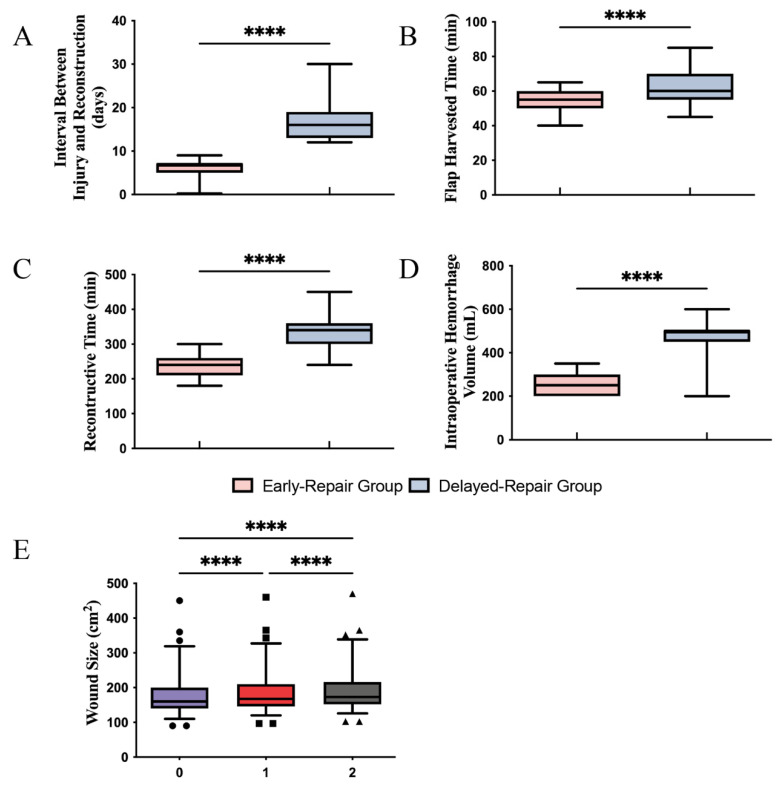
The perioperative conditions of early- and delayed-repair group. (**A**) The interval between injury and reconstruction was significantly longer in the delayed-repair group compared to early-repair group. (**B**–**D**) The surgical difficulty for delayed reconstruction was notably increased, which was reflected by prolonged flap harvest and reconstructive times, and increased intraoperative hemorrhage volumes in the delayed-repair group. (**E**) The wound size was gradually enlarged due to multiple debridements, with wound area increasing by an average of 8.20% after two debridements. **** *p* < 0.0001.

Due to the condition of the wounds and different surgical patterns conducted, the mean reconstructive intervals, the flap harvest time, and the reconstructive procedure times were significantly prolonged in the delay-repair group (16.46 ± 4.09 days, 63.34 ± 9.85 min, and 334.2 ± 48.85 min) compared to the early-repair group (6.15 ± 1.82 days, 54.28 ± 8.31 min, and 239.1 ± 32.11 min) (*p* < 0.0001, Figure 4A–C). The intraoperative blood loss in the delayed-repair group was also significantly greater than in the early-repair group. Specifically, the mean blood loss was 481.5 ± 84.99 mL in the delayed-repair group and 256.3 ± 47.90 mL in the early-repair group (*p <* 0.0001, Figure 4D). In addition, we observed an increase in wound size in the delayed-repair group with every debridement, with the wound area increasing by an average of 8.20% after two debridements (*p <* 0.0001, Figure 4E).

The incidence and severity of flap complications were much higher in the delayed-repair group than in the early-repair group (*p* = 0.007). Specifically, the incidences of overall flap complications and total flap necrosis were 2.34 and 4.45 times higher in the delayed-repair group. The higher flap complication incidence was due to more risk factors in the delayed-repair group, as presented in Table 2. No statistical difference was observed in donor site morbidities across both groups (Table 2).

**Figure 5:**
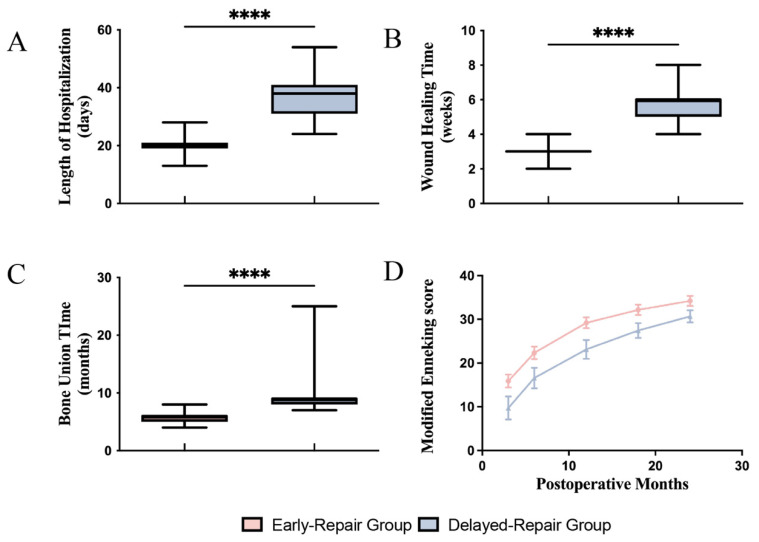
The short- and long-term follow-up of early- and delayed-repair group. (**A**) The LoS of delayed-repair group was 1.71 times longer than the early-repair group. (**B**,**C**) The wound healing time and bone re-union time were significantly longer in the delayed-repair group than the early-repair group. (**D**) All cases gained satisfying functional results by 24 months postoperative. However, the early-repair group gained better functional recovery throughout the follow-ups. LoS, length of hospitalization; **** *p* < 0.0001.

Delayed repair also resulted in prolonged hospital stays and healing processes. The LoS was 36.39 ± 8.09 days in the delayed-repair group, which was 1.71 times longer than the early-repair group, in which the LOS was 21.32 ± 3.77 days (*p <* 0.0001, Figure 5A). Wound healing time and bone healing time were significantly longer in the delayed-repair group (5.61 ± 1.17 weeks and 9.08 ± 2.54 months, respectively) than in the early-repair group (3.21 ± 0.48 weeks and 5.71 ± 0.96 months respectively) (*p* < 0.0001, Figure 5B,C).

The overall late-complication rate was also significantly higher in the delayed-repair group (11.45% in the delayed-repair group and 4.20% in the early-repair group, *p* = 0.010). In addition, 11 cases in the delayed-repair group (4.85%) underwent additional operations on the bone, while no bone surgery was needed in the early-repair group (*p* = 0.003, Table 2). Bone grafting was used for the nonunion of fracture, and Ilizarov bone transport was used to solve the bone defect. All fractures achieved complete bone healing.

All cases gained satisfying functional results by 24 months postoperative. However, the early-repair group showed better functional recovery than the delayed-repair group throughout follow-ups, according to modified Enneking scoring (Figure 5D).

## 4. Discussion

The optimal timing of soft-tissue reconstruction in Gustilo IIIb/IIIc fractures is important and has become a controversial subject. This study analyzed the outcomes of different reconstruction intervals in Gustilo IIIB tibial fractures. We reported shorter flap harvest and reconstructive times, less intraoperative bleeding, lower flap complication rates, shorter LoS, faster wound and bone healing, and lower late-complication rates in the early-repair group than in the delayed-repair group. Satisfactory function restoration was achieved in all groups, although the early-repair group showed better functional recovery than the delayed-repair group throughout the follow-ups.

In accordance with the previous literature, our data showed that early flap reconstruction using an orthoplastic approach achieved better outcomes than the delayed reconstruction in Gustilo IIIb/IIIc fractures by integrating the unique strengths of orthopeadic and plastic surgeons. Back in 1986, Godina et al. first highlighted the benefits of early reconstruction [11]. Recently, Qiu et al. demonstrated that reconstruction performed within 72 h of injury reduced the rates of flap total or partial failure and infection [26]. A meta-analysis of studies by Haykal et al. also found the rates of free flap failure and infection were significantly lower when reconstruction was performed within 72 h [27]. While several sources in the literature have concurred with Godina regarding the advantages of early reconstruction, optimal timing varied somewhat. Byrd et al. recommended early coverage within 5 days, while Yaremchuk et al. advocated that microsurgical reconstruction be performed between 1 and 2 weeks after injury [12,28,29]. Similarly, Francel et al. observed 72 patients with Gustilo IIIB tibial fractures within 15 days to have a major complications risk of 3.6% [30]. A recent retrospective review included 358 soft-tissue free flaps indicated that the ideal early period of reconstruction can be safely extended for up to 10 days after injury without adversely effecting outcomes [31]. Despite these slight differences in optimal timing, the data from all of those works emphasized the superiority of early flap reconstruction.

On the other hand, a growing body of literature has proposed that delayed reconstruction does not lead to worse outcomes. Kolker et al. reported no bearing on outcomes in reconstructions for lower-extremity injuries performed within 21 days compared to those performed within 22 to 60 days, and even more than 60 days, after injury [32]. Hill et al. also demonstrated that no statistically significant differences in flap failure were observed for reconstructions performed within 30 days versus 31 to 90 days after injury [24]. Similarly, Starnes-Roubaud et al. reported no difference in flap failure, osteomyelitis, or bone union between free flap coverages performed within 15 days compared to those performed 15 days after lower-extremity injuries [33]. Recently, Arslan et al. indicated no significant difference in flap failure and complication rates between free flap reconstructions performed after 10 to 29 days, and more than 30 days, in open lower-extremity fractures [25]. Some papers have also confirmed the reliability of delayed reconstruction. Karanas et al. reported no flap loss and a case of late osteomyelitis in 14 lower-extremity reconstructions undertaken between 30 days and 3 months from injury [22]. These findings, contrary to our results, also emphasize the fact that the optimal timing of free flap reconstruction in complex extremity injuries remains an important and controversial topic.

Despite the controversy, our data validate the merits of early reconstruction in lower-extremity open fractures. The delayed reconstructions were more difficult to perform due to the extensive fibrosis involving not only tendons and muscles but also the neurovascular bundles in the delayed-repair group compared to the early-repair group, reflected by prolonged harvest and reconstructive times and greater intraoperative bleeding (Figure 4B–D) [11]. Furthermore, the rates of wound infection and coagulation abnormity were also higher in the delayed-repair group than in the early-repair group because of inadequate debridement (Table 1). Given the high contamination, the importance of thorough debridement in the management of open fracture has been emphasized by numerous publications [34,35,36]. Briefly, this process involves cleaning the surface of wound to remove gross contamination and assess the injury, then adequately extending the wound to explore all the zones of injury systematically in turn, from superficial to deep (skin, fat, muscle, bone) and from the periphery to the centre of the wound, to remove all devitalized tissue. All free bone fragments and bones failing the “tug test” should be removed, while the neurovascular bundles should be preserved as much as possible. Identifying the optimal degree of debridement of open fractures remains a challenge for surgeons: inadequate debridement leads to deep infections, while radical debridement causes bone nonunion. Ricci et al. indicated aggressive bone debridement may lead to segmental bone loss, delayed bone union, and additional surgical procedures to promote union [37]. A more severe infection has a higher risk of insufficient debridement, which not only increases the number of surgical procedures but also delivers worse outcomes in soft-tissue coverage. In addition, in many open fractures, it is difficult to distinguish whether tissues are devitalized or viable at the time of initial debridement [38]. Handling the dilemma of the degree of debridement demands extensive clinical experience; the BOASTs upgrade the qualifications of practitioners who perform debridement procedures from senior plastic and orthopaedic surgeons to consultants in orthopaedic and plastic surgery [10]. However, the majority of emergency debridement in China is performed by junior surgeons, where inadequate debridement leads to wound infection and worse wound conditions, which leads to additional debridement and additional VSD coverage. On one hand, additional debridement is related to the higher risk of polymicrobial posttraumatic osteomyelitis; on the other hand, in our experience, continued drainage by additional VSD coverage also leads to a higher risk of bone nonunion due to the loss of albumins and periosteal damage [39]. Even in cases of thorough initial debridement, delayed repair with prolonged application of VSD delivered worse outcomes, which can be partially attributed to the loss of proteins, immunoglobulins, and electrolytes in exudates due to continuous suction [40,41]. The VSD created more wound-associated protein loss than burn wounds. In addition, despite the fact that the drainage of oedema by VSD reduced the size of the wound, multiple debridements expanded the wound gradually in the delayed-repair group, in which debridement was performed more than three times (Figure 4E). The delayed wound closure popular in China, as well as a lack of an orthoplastic center, make the reconstruction of severe limb injury more complicated and more difficult [42].

This study was a retrospective study with inherent limitations, and the cohort included 394 participants. In particular, the injury conditions and the degree of initial debridement of referral patients were evaluated through hospitalization data; the lack of an initial assessment of primary injury conditions may lead to selective bias. We recommend the early reconstruction of Gustilo IIIb fractures to accelerate wound healing and bone reunion, as well as early referral to a higher-level hospital with orthoplastic capabilities after an aggressive and thorough initial debridement carried out by senior surgeons.

## 5. Conclusions

In summary, early repair with free flaps performed within two instances of debridement had superior outcomes with reduced difficulty of reconstruction, lower risks of flap necrosis and complications, shorter LoS, accelerated wound and bone healing, and decreased rates of overall late complications compared with delayed reconstruction after multiple debridements, in accordance with Godina’s findings. Early soft-tissue reconstruction after thorough debridement was an effective and reliable option for severe lower-extremity open fractures. We recommend early referral to a higher-level hospital with orthoplastic capabilities after an aggressive and thorough initial debridement carried out by senior surgeons, as well as the early reconstruction of Gustilo IIIb fractures to accelerate wound healing and bone reunion.

## Figures and Tables

**Table 1:** Demographic data.

Variable	Early-Repair Group (0–1, N = 167)	Delayed-Repair Group (≥2, N = 227)	*p* Value ^#^
Age (year)	41.9 ± 13.5	40.2 ± 11.8	0.470
Gender			0.836
Male	98	136	
Female	69	91	
BMI			0.370
Normal	114	165	
Abnormal	53	62	
Alcohol History			0.729
Yes	46	58	
No	121	169	
Smoking History			0.544
Yes	41	49	
No	126	178	
Chronic diseases			0.781
Yes	28	35	
No	139	192	
Cause of injury			0.500
Traffic accident	108	134	
Crash injury	42	64	
Agricultural accidents	17	29	
Source of the patient			0.001
Emergency	132	25	
Referral within 1 week	35	88	
Referral after 1 week	0	114	
Injury location			0.760
Proximal	11	21	
Proximal/middle	19	27	
Middle	28	35	
Middle/distal	37	57	
Distal	72	87	
Wound infection			0.001
Yes	19	182	
No	148	45	
Coagulation factors			0.001
Normal	134	28	
abnormal	33	199	
Defect size (cm^2^)	180.4 ± 49.6	183.4 ± 51.5	0.558
Soft tissue repair			0.556
ALTP	67	98	
LD	53	76	
Other *	47	53	
Flap size (cm^2^)	197.2 ± 50.6	199.0 ± 51.2	0.731

Data represent mean ± standard deviation or number. BMI, body mass index; ^#^ Two-sided Fisher’s exact test. ALTP, anterolateral thigh perforator flap; LD, latissimus dorsi musculocutaneous flap. * Combination to repair the wound.

**Table 2:** Short- and long-term follow-up results.

Variable	Early-Repair Group (0–1, N = 167)	Delayed-Repair Group (≥2, N = 227)	*p* Value ^#^
Flap complications	11/156	35/192	0.007
Total flap necrosis	2 ^1^	12 ^2^	
Partial flap necrosis	6	15	
Subcutaneous ulcer	3	8	
Factors of flap necrosis			
Vascular crisis	6	15	-
Infection	3	11	
Hematoma	2	9	
Donor site morbidity			
Delayed wound healing	3	6	0.739
Chronic infection ^&^	7	26	0.010
Reoperation *	0	11	0.003
Nonunion of fracture	0	5	
Bone defects	0	6	

^&^ deep infection/ osteomyelitis. * Bone grafting was used for nonunion of fracture; Ilizarov bone transport was used to solve the bone defect. ^1^ 2 DIEP were harvested for second reconstruction. ^2^ 7 DIEP, 3 contralateral LD and 2 contralateral ALTP were harvested for second reconstruction. ^#^ Two-sided Fisher’s exact test. DIEP: deep inferior epigastric artery flap; ALTP: anterolateral thigh perforator flap; LD: latissimus dorsi musculocutaneous flap.

## Data Availability

The data presented in this study are available on request from the corresponding author. The data are not publicly available due to privacy.
